# Incorporation of a hinge domain improves the expansion of chimeric antigen receptor T cells

**DOI:** 10.1186/s13045-017-0437-8

**Published:** 2017-03-13

**Authors:** Le Qin, Yunxin Lai, Ruocong Zhao, Xinru Wei, Jianyu Weng, Peilong Lai, Baiheng Li, Simiao Lin, Suna Wang, Qiting Wu, Qiubin Liang, Yangqiu Li, Xuchao Zhang, Yilong Wu, Pentao Liu, Yao Yao, Duanqing Pei, Xin Du, Peng Li

**Affiliations:** 1 Key Laboratory of Regenerative Biology, South China Institute for Stem Cell Biology and Regenerative Medicine, Guangzhou Institutes of Biomedicine and Health, Chinese Academy of Sciences, Guangzhou, 510530 China; 2 Guangdong Provincial Key Laboratory of Stem Cell and Regenerative Medicine, South China Institute for Stem Cell Biology and Regenerative Medicine, Guangzhou Institutes of Biomedicine and Health, Chinese Academy of Sciences, Guangzhou, 510530 China; 3 State Key Laboratory of Respiratory Disease, Guangzhou Institutes of Biomedicine and Health, Chinese Academy of Sciences, Guangzhou, 510530 China; 4 Department of Hematology, Guangdong General Hospital/Guangdong Academy of Medical Sciences, Guangzhou, 510080 Guangdong China; 5 InVivo Biomedicine Co. Ltd, Guangzhou, 510000 China; 6 Institute of Hematology, Medical College, Jinan University, Guangzhou, 510632 China; 7 Guangdong Lung Cancer Institute, Medical Research Center, Guangdong General Hospital, Guangdong Academy of Medical Sciences, Guangzhou, China; 8 Wellcome Trust Sanger Institute, Hinxton, Cambridge, CB10 1HH England, UK

**Keywords:** CAR T cell, Hinge domain, Expansion, CD4+ T cell, CD19, Mesothelin

## Abstract

**Background:**

Multiple iterations of chimeric antigen receptors (CARs) have been developed, mainly focusing on intracellular signaling modules. However, the effect of non-signaling extracellular modules on the expansion and therapeutic efficacy of CARs remains largely undefined.

**Methods:**

We generated two versions of CAR vectors, with or without a hinge domain, targeting CD19, mesothelin, PSCA, MUC1, and HER2, respectively. Then, we systematically compared the effect of the hinge domains on the growth kinetics, cytokine production, and cytotoxicity of CAR T cells in vitro and in vivo.

**Results:**

During in vitro culture period, the percentages and absolute numbers of T cells expressing the CARs containing a hinge domain continuously increased, mainly through the promotion of CD4+ CAR T cell expansion, regardless of the single-chain variable fragment (scFv). In vitro migration assay showed that the hinges enhanced CAR T cells migratory capacity. The T cells expressing anti-CD19 CARs with or without a hinge had similar antitumor capacities in vivo, whereas the T cells expressing anti-mesothelin CARs containing a hinge domain showed enhanced antitumor activities.

**Conclusions:**

Hence, our results demonstrate that a hinge contributes to CAR T cell expansion and is capable of increasing the antitumor efficacy of some specific CAR T cells. Our results suggest potential novel strategies in CAR vector design.

**Electronic supplementary material:**

The online version of this article (doi:10.1186/s13045-017-0437-8) contains supplementary material, which is available to authorized users.

## Background

In the last 5 years, chimeric antigen receptor (CAR) T cells have emerged from bench to bedside and made headlines in clinical trials at a number of academic institutions [1–4]. CARs are recombinant receptors that specifically target tumor-surface antigens. Once the CARs are transfected into T cells, the cells acquire supraphysiologic properties and act as “living drugs” [5, 6]. Multiple iterations of CARs have been developed, mainly focusing on intracellular signaling modules, which are deemed crucial for CAR design [7, 8]. To achieve appropriate costimulatory signals so as to activate effector T cells, improve response, and prolong persistence, many different kinds of costimulatory receptors can be incorporated (e.g., CD28 [9, 10], 4-1BB [11, 12], OX40 [13], ICOS [14], and CD27 [15]), alone or in tandem [16]. However, the effect of non-signaling extracellular modules, such as hinge and TM domains, on the proliferation of the transduced T cells and therapeutic efficacy of CARs remains largely unclear [17].

A hinge domain is a structure between the targeting moiety and the T cell plasma membrane [18]; these sequences are generally derived from IgG subclasses (such as IgG1 and IgG4), IgD and CD8 domains, of which IgG1 has been most extensively used [19–21]. Currently, studies of the hinge domain mainly focus on the following four aspects: (1) reducing binding affinity to the Fcγ receptor, thereby eliminating off-target activation [19, 21]; (2) enhancing the single-chain variable fragment (scFv) flexibility, thereby relieving the spatial constraints between tumor antigens and CARs, in turn promoting synapse formation between the CAR T cells and target cells; for example, to overcome steric hindrance in MUC1-specific CAR, a flexible and elongated hinge of the IgD isotype can be inserted [20]; (3) reducing the distance between an scFv and the target epitope, for example, anti-CD22 CAR needs a hinge domain to exert optimal cytotoxicity [22]; and (4) facilitating the detection of CAR expression using anti-Fc reagents. Nevertheless, the influences of the hinge domain on CAR T cell physiology are not well understood.

To better understand the effect of the hinge domain on CAR T cells, we generated two versions of CARs, with or without a hinge domain, targeting CD19, mesothelin, PSCA (prostate stem cell antigen), MUC1, and HER2 (human epidermal growth factor receptor 2), respectively [23–31]. We systematically compared the effect of the hinge domains on the growth kinetics, cytokine production, and cytotoxicity of CAR T cells in vitro and in vivo. We revealed that the incorporation of a hinge into CAR constructs can substantially increase the CAR T cell percentage during the in vitro culture period, enhance the invasiveness of CAR T cells. In addition, we found that anti-CD19 CAR T cells with or without a hinge domain have similar abilities to eliminate leukemia cells, whereas a hinge domain can enhance the in vivo antitumor activity of anti-mesothelin CAR T cells.

## Methods

### Cells and culture conditions

NALM6-GL (acute lymphoblastic leukemia line, stably transfected with GFP and luciferase) and A549-GL (human lung cancer cell line, stably transfected with GFP and luciferase) cell lines were cultured in RPMI-1640 (Gibco, Life Technologies). HEK293T cells used for lentivirus production were cultured with DMEM (Gibco, Life Technologies). DMEM and RPMI-1640 media were supplemented with 10% heat-inactivated FBS (Gibco, Life Technologies), 10 mM of HEPES, 100 U/ml of penicillin, 100 μg/ml of streptomycin, and 2 mM of l-glutamine (Gibco, Life Technologies).

Human peripheral blood mononuclear cells (PBMCs) from healthy donors were obtained from Guangdong General Hospital, after obtaining informed consent and for research use only. Pan T cells were enriched from the PBMCs using a “Pan T cell isolation kit” (Miltenyi Biotec, Germany). CD8+ T cells were positively isolated from the Pan T cells using “CD8 microbeads” (Miltenyi Biotec, Germany); the unlabeled cells that passed through the column were collected, representing the CD4+ T cells.

### Construction of chimeric antigen receptors (CARs)

CD19.28z, Meso.28z, HER2.28z, and PSCA.28z CARs were constructed by linking sequences from a signal peptide derived from GM-CSF (GenBank; AAA58735.1, aa 1–19) to the corresponding antigen-specific single-chain variable fragment (scFv); these CARs do not have a spacer domain. Anti-CD19 scFv derived from FMC63 monoclonal antibody, anti-Mesothelin scFv derived from SS1 monoclonal antibody, anti-HER2 scFv derived from FRP5 monoclonal antibody, and PSCA scFv derived from humanized 1G8 monoclonal antibody. The CARs were codon-optimized and chemically synthesized using Genscript. A spacer containing a hinge domain and a CH3 domain derived from human IgG4 (GenBank; AAC82527.1, aa 98–329) was included in PSCA-H.28z CAR; an additional IgD hinge spacer was included in MUC1-H.28z CAR. The scFv of anti-MUC1 CAR derived from HMFG2 monoclonal antibody. The resulting products were sub-cloned into a Pwpxld-based lentiviral backbone plasmid encoding the transmembrane and intracellular domains of CD28 (aa 153–220) and the intracellular domain of CD3-ζ (aa 52–163). CD19-H.28z CAR and Meso-H.28z CAR were constructed by overlapping PCR, adding the hinge domain and the CH3 of IgG4 to the 3′ end of the scFv.

### Lentiviral production and transduction

Pwpxld (encoding the CARs or GFP), Pspax2 (expressing the three required lentiviral proteins), and PMD.2G (encoding the lentiviral envelope protein) were transfected into HEK293T cells with PEI reagent (Life Technologies). The lentiviral supernatants were collected 48 and 72 h after transfection and were passed through a 0.4 μm filter. Human Pan T cells, CD8+ T cells, or CD4 T cells were stimulated with microbeads loaded with anti-human CD3, anti-human CD2, and anti-human CD28 antibodies (Miltenyi Biotec, Germany) in a 3:1 bead:cell ratio for 72 h and cultured in RPMI 1640, supplemented with 10% FBS, 10 mM of HEPES, 100 U/ml of penicillin, 100 μg/ml of streptomycin, 2 mM of l-glutamine, r-human IL-2 (300 IU/ml, PeproTech, CT, USA), and r-human IL-15 (5 ng/ml, PeproTech, CT, USA). On day 3 after cell activation, the activated T cells were transduced with lentiviral supernatants and Polybrene 8 μg/ml (Takara) once; then, the cells were washed with PBS 3 times to completely remove any residual lentiviral supernatants. The cells were then resuspended in complete medium to achieve the expansion. The microbeads were removed on day 5. Fresh medium was added every 2 days to maintain an appropriate cell density ranging from 5 × 10^5^ to 1 × 10^6^ cells/ml.

### Flow cytometry

All of the samples were analyzed with an LSR Fortessa or C6 (BD Bioscience), and the data were analyzed using FlowJo software. CAR T cells were detected by GFP, and T cell phenotypes were evaluated via CD3 PE-cy7 (clone OKT3, eBioscience), CD3 BV421 (clone UCHT1, BD), CD8a PE (clone HT8a, eBioscience), CD8a PE-cf594 (clone RPA-T8, BD), and CD4 APC (clone OKT4, eBioscience). All FACS plots representing the CAR T cell phenotypes were gated on CD3 and GFP double positive cells.

### In vitro tumor-killing assays and cytokine-release assays

The target leukemia cells, NALM6-GL, were co-cultured with GFP T, 19.28z, or 19-H.28z T cells at the indicated E:T ratios in triplicate in U-bottomed 96-well plates for 18 h; A549-GL cells were co-cultured with GFP T, Meso.28z T, or Meso-H.28z T cells, each well of 96-well plates contained 200 μl supernatants. One hundred microliter supernatants from the wells with E:T ratios of 1:1 were collected and used for detecting the concentrations of IL-2 and IFN-γ using an ELISA kit (eBioscience). The luciferase substrate d-luciferin (potassium salt, 150 μg/ml, Cayman Chemical, USA) was added to a 100 μl/well, and the viability of the target cells was monitored by a microplate reader at a 450-nm excitation wavelength. The background luminescence was negligible (<1% of the signal from the wells with only target cells); therefore, the target cell viability (%) was calculated as (experimental signal/maximal signal) × 100%, and the killing percentage was calculated as 100% − viability percentage.

### Transwell cell migration assay

For the 19.28z T and 19-H.28z T cells, Nalm6 cell lysates were used as a chemoattractant in the lower chamber, while for the Meso.28z T and Meso-H.28z T cells, A549 cell lysates were used as a chemoattractant in the lower chamber. The T cells were cultured in an insert coated with Matrigel for 24 h, and the cells that transmigrated to the lower chamber were counted by flow cytometry. The percentage of migration was calculated as follows: (CAR T cells migrating through the Matrigel chamber membrane/total CAR T cells in insert membrane before assay begin) × 100.

### In vivo studies

NSI mice (NOD-scid-IL2Rg^−^/^−^mice, Guangzhou Institutes of Biomedicine and Health (GIBH)), aged 6–12 weeks were used to construct the xenograft tumor mouse models. All of the animal studies were carried out in accordance with instructional guidelines from the China Council on Animal Care and under protocols approved by the guidelines of the Ethics Committee of Animal Experiments at GIBH. Equivalent numbers of male and female mice were used. The NALM6-GL leukemia lines were intravenously inoculated into 2 × 10^5^ cells, and the A549-GL carcinoma lines were inoculated into 5 × 10^5^ cells subcutaneously (flank). The mice then received an adoptive transfer of CAR T cells intravenously 3–14 days later, as indicated in the individual experiments. To control the differences in transduction efficiency, non-transduced T cells were supplemented to ensure that both the number of CAR+ T cells, and the total number of T cells remained constant across all CAR T cell groups. The leukemia burden was evaluated using a cooled CCD camera system (IVIS 100 Series Imaging System, Xenogen, Alameda, CA, USA). The mice were injected intraperitoneally with d-luciferin firefly potassium salt at 75 mg/kg and then imaged 5 min later with an exposure time of 30 s. Quantification of the total and average emissions was performed using Living Image software (Xenogen).

### Statistics

All graphs report the mean ± SEM. All statistical analyses were performed with Prism software version 6.0 (GraphPad). The statistical significance of all data was calculated using an unpaired Student’s *t* test with the Bonferroni correction for multiple comparisons, where applicable. *P* < 0.05 was considered significant and is designated with an asterisk in all figures.

## Results

### Hinge incorporation can promote the growth of CAR T cells

**Fig. 1:**
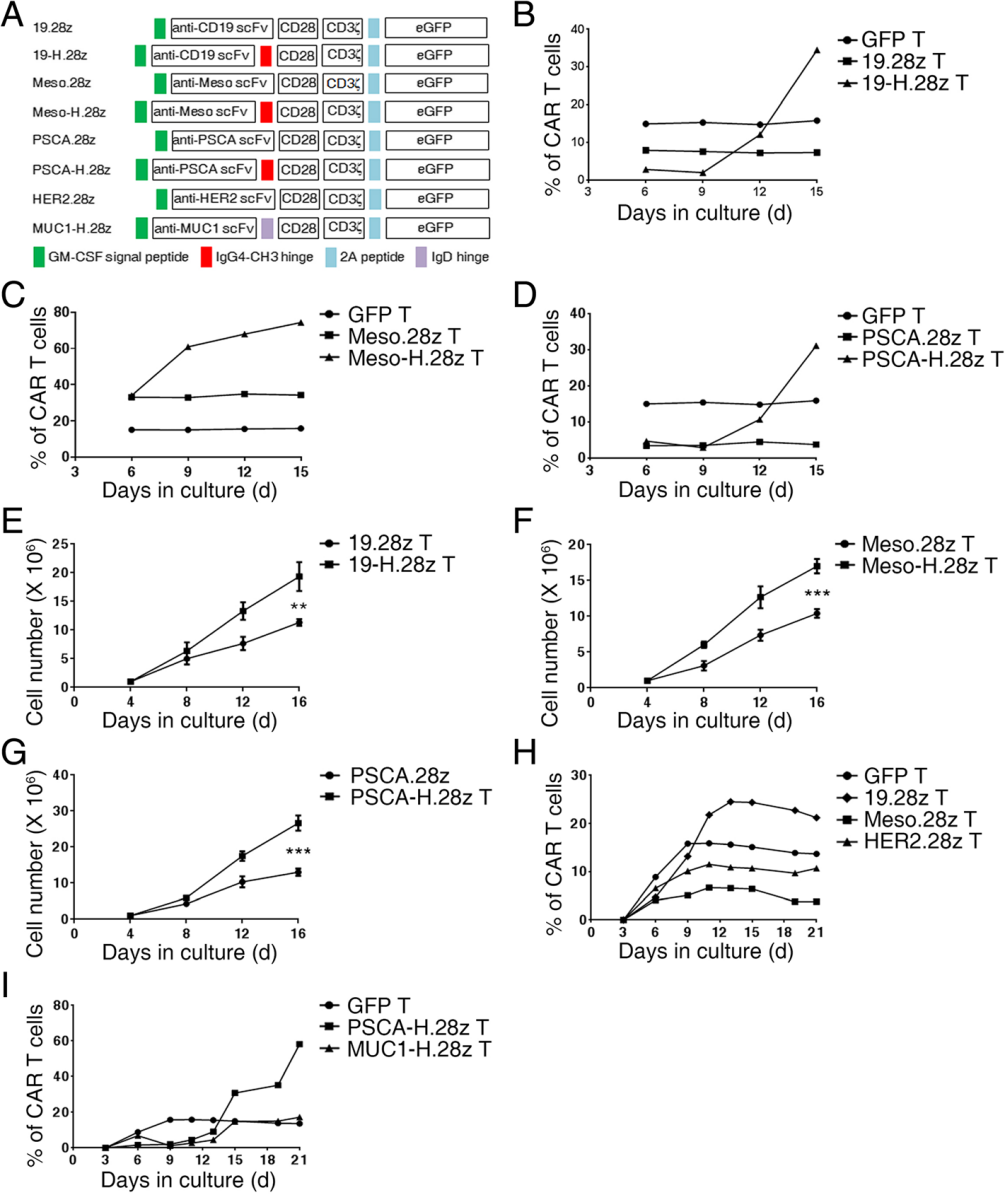
Hinge incorporation can promote the expansion of CAR T cells. **a** Schematic representation of CAR constructs specific for different antigens, and with or without a hinge domain. Flow cytometric analysis of the percentage of (**b**) 19.28z, 19-H.28z T cells, (**c**) Meso.28z, Meso-H.28z T cells, (**d**) PSCA.28z, PSCA-H.28z T cells, and GFP control T cells from day 6 to 15 during the in vitro culture period. The data are representative of independent experiments verified with cells from over three individual healthy human donors. Total cell number of (**e**) 19.28z T and 19-H.28z T cells, (**f**) Meso.28z T and Meso-H.28z T cells, (**g**) PSCA.28z T, and PSCA-H.28z T cells from day 4 to day 16 during the in vitro culture period. *Error bars* denote the SEM, and the results were compared through an unpaired *t* test. *P < 0.05, **P < 0.01, and ***P < 0.001. Flow cytometric analysis of the percentage of (**h**) 19.28z T, Meso.28z T, HER2.28z T cells, (**i**) PSCA-H.28z T, MUC1-H.28z T, and GFP control T cells from day 6 to 21 during the in vitro culture period. The data are representative of independent experiments verified with cells from over three individual healthy human donors

To characterize the functionality of hinges in CAR vectors, we constructed a series of lentiviral vectors for CAR production that contained the same transmembrane and intracellular domains (CD28 costimulatory receptor in tandem with CD3ζ) but different scFv-binding and hinge domains, tagged with GFP (Fig. 1a). We then transduced these CAR vectors into human primary T cells and monitored the growth of the CAR T cells from day 6 post-transfection. Interestingly, we found that the percentages of CAR T cells were greater when the CAR vectors contained a hinge domain, regardless of the scFv. For example, the percentages of 19-H.28z and Meso-H.28z T cells increased from 3.0 to 40.7% (Fig. 1b; Additional file 1: Figure S1) and from 34 to 74.3% (Fig. 1c; Additional file 1: Figure S1), respectively. In addition, the percentages of PSCA-H.28z T cells increased from 4.7 to 31.1% (Fig. 1d; Additional file 1: Figure S1) and from 1.6 to 58.2% (Fig. 1i). Similarly, the percentages of MUC1-H.28z T cells also increased from 6.9 to 17.3%, although the MUC1-H.28z CAR was designed with an IgD hinge, which indicates that the ability to increase the CAR T cell percentage is not restricted to IgG4-CH3 hinges (Fig. 1i). The absolute number of CAR T cells also significantly increased (Fig. 1e–g). In contrast, the percentages of CAR T cells without a hinge domain tended to be stable throughout the in vitro expansion process (Fig. 1b–d; Additional file 1: Figure S1), and their total cell numbers were less than those of CAR T cells containing a hinge domain (Fig. 1e–g). Taken together, these results demonstrate that the incorporation of a hinge domain can promote the growth of CAR T cells in vitro.

### Hinge incorporation mainly promotes CD4+ CAR T cell expansion

**Fig. 2:**
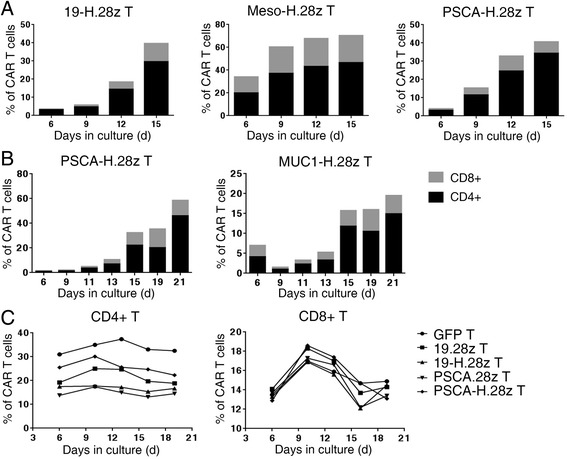
Hinge incorporation mainly promotes CD4+ CAR T cell expansion. **a** Flow cytometric analysis of the percentage of CD4+ and CD8+ 19-H.28z T (*left*), Meso-H.28z T (*middle*), and PSCA-H.28z T (*right*) during the in vitro culture period. *Black box* represents CD4+ T cell and *gray box* represents CD8+ T cells. The data are representative of independent experiments verified with cells from over three individual healthy human donors. **b** Flow cytometric analysis of the percentage of CD4+ and CD8+ PSCA-H.28z T (*left*) and MUC1-H.28z T cells (*right*) during the in vitro culture period. *Black box* represents CD4+ T cell and *gray box* represents CD8+ T cells. The data are representative of independent experiments verified with cells from over three individual healthy human donors. **c** Flow cytometric analysis of the percentage of CD4+ and CD8+ GFP T, 19.28z T, 19-H.28z T, PSCA.28z T, and PSCA-H.28z T cells when CD4+ T and CD8+ T cells were isolated and cultured them separately in vitro. The data are representative of independent experiments verified with cells from over three individual healthy human donors

To identify which T cell subsets were most responsive to the incorporation of a hinge domain, we monitored the ratios of CD4+ and CD8+ CAR T cells in culture and found that the percentages of CD4+ 19-H.28z T and CD4+ Meso-H.28z T cells increased from 3.13 to 29.6% (Fig. 2a, left; Additional file 2: Figure S2) and from 19.9 to 46.5% (Fig. 2a, middle), respectively. In addition, the percentages of CD4+ PSCA-H.28z T cells increased from 3.06 to 34.3% (Fig. 2a, right) and from 0.92 to 46% (Fig. 2b, left). The CD4+ MUC1-H.28z T cells also increased from 4.14 to 14.9% (Fig. 2b, right). However, the influence of the hinge domain on the percentages of CD8+ CAR T cells was less pronounced, as the percentages of CD8+ 19-H.28z T and CD8+ Meso-H.28z T cells increased from 0.4 to 10.5% (Fig. 2a, left; Additional file 2: Figure S2) and from 14.1 to 23.8% (Fig. 2a, middle), respectively. In addition, the CD8+ PSCA-H.28z T cells increased from 0.92 to 6.34% (Fig. 2a, right) and from 0.67 to 12.6% (Fig. 2b, left). The CD8+ MUC1-H.28z T cells also increased from 2.85 to 4.61% (Fig. 2b, right). The percentage of CAR T cells without a hinge domain tended to be stable throughout the in vitro culture period (Additional file 3: Figure S3). Interestingly, when we isolated CD4+ T and CD8+ T cells and cultured them separately in vitro, the percentages of CAR T cells with or without a hinge domain all tended to be stable in both CD4+ T and CD8+ T cells, consistent with the GFP control T cells (Fig. 2c). This suggests that the use of a hinge to enhance CD4+ CAR T cell expansion requires the co-participation of CD8+ CAR T cells. In summary, hinge incorporation mainly promotes CD4+ CAR T cell expansion during the in vitro culture period.

### Hinge incorporation can enhances migratory capacity of CAR T cells

**Fig. 3:**
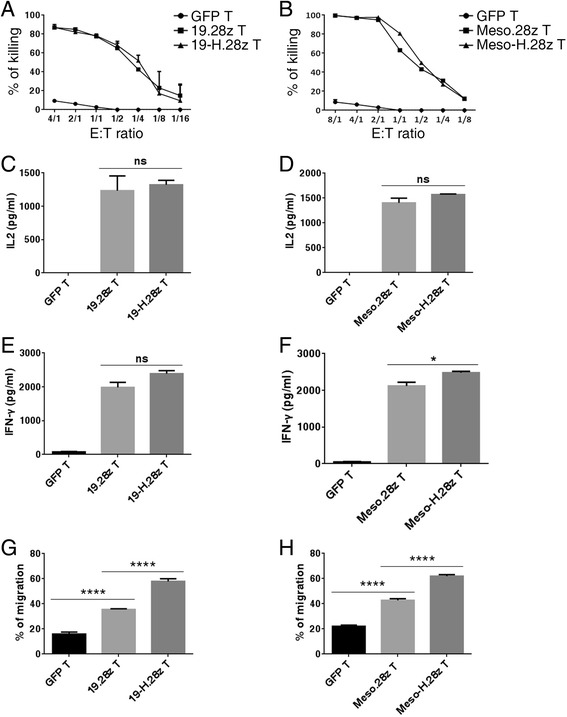
A hinge enhances the migratory capacity of CAR T cells. Cytotoxicity of (**a**) 19.28z T, 19-H.28z T, and control GFP T cells after co-culture with CD19+ cell line (NALM6-GL) for 24 h, (**b**) Meso.28z T, Meso-H.28z T, and control GFP T cells after co-culture with mesothelin + cell line (A549-GL) for 24 h. E:T ratios are the ratios of the absolute number of CAR T cells to the target cells. The GFP percentages of the CAR T cells were equalized using non-transduced T cells from the same donor. *n* = 3 replicates per point; the data are representative of independent experiments verified with cells from over three individual healthy human donors. IL2 production of (**c**) 19.28z T, 19-H.28z T, and control GFP T cells after co-culture with CD19+ cell line (NALM6-GL) for 24 h, (**d**) Meso.28z T, Meso-H.28z T, and control GFP T cells after co-culture with mesothelin + cell line (A549-GL) for 24 h. IFN-γproduction of (**e**) 19.28z T, 19-H.28z T, and control GFP T cells after co-culture with CD19+ cell line (NALM6-GL) for 24 h, (**f**) Meso.28z T, Meso-H.28z T, and control GFP T cells after co-culture with mesothelin + cell line (A549-GL) for 24 h. CAR T cells were co-cultured with the targeted tumor cells at a 1:1 E: T ratio. *n* = 3 replicates per point; the data are representative of independent experiments verified with cells from over three individual healthy human donors. *Error bars* denote the SEM, and the results were compared through an unpaired *t* test. *P < 0.05, **P < 0.01, and ***P < 0.001. Transwell cell migration assay indicated an improved capacity of (**g**) 19-H.28z T cells, (**h**) Meso-H.28z T cells to transmigrate Matrigel. For the 19.28z T and 19-H.28z T cells, Nalm6 cell lysates were used as a chemoattractant in the lower chamber, while for the Meso.28z T and Meso-H.28z T cells, A549 cell lysates were used as a chemoattractant in the lower chamber. The T cells were cultured in an insert coated with Matrigel for 24 h, and the cells that transmigrated to the lower chamber were counted by flow cytometry. The percentage of migration was calculated as follows: (CAR T cells migrating through the Matrigel chamber membrane/total CAR T cells in insert membrane before assay begin) × 100. *n* = 3 replicates per point; the data are representative of independent experiments verified with cells from over three individual healthy human donors. *Error bars* denote the SEM, and the results were compared through an unpaired *t* test. *P < 0.05, **P < 0.01, ***P < 0.001, and ****P < 0.0001

To study whether the incorporation of a hinge domain affects the cytotoxicity of CAR T cells, we compared the killing capacities of anti-CD19 and anti-mesothelin CARs with and without a hinge. Both 19.28z T and 19-H.28z T cells efficiently lysed the NALM6-GL (Fig. 3a), indicating that the killing capacities of these two CARs were similar. Similarly, there were no significant differences between the lysis capacities of Meso.28z T and Meso-H.28z CAR T cells (Fig. 3b). For cytokine production, both 19-H.28z T and Meso-H.28z T cells produced similar levels of IL2 and IFN-γ compared with their hinge-free counterparts (Fig. 3c–f). Next, we compared the migratory capacity of GFP T, 19.28z T, and 19-H.28z T cells, using NALM6 cell lysate as a chemoattractant in the lower chamber of the transwell plate. Interestingly, we found that the 19-H.28z T cells transmigrated the Matrigel more efficiently than the 19.28z T cells (Fig. 3g). Similar results were also obtained in the Meso-H.28z T cells (Fig. 3h), suggesting that hinge incorporation enhanced the migratory and invasion capabilities of CAR T cells.

### 19-H.28z T and 19.28z T cells have comparable anti-tumor efficacy in vivo

**Fig. 4:**
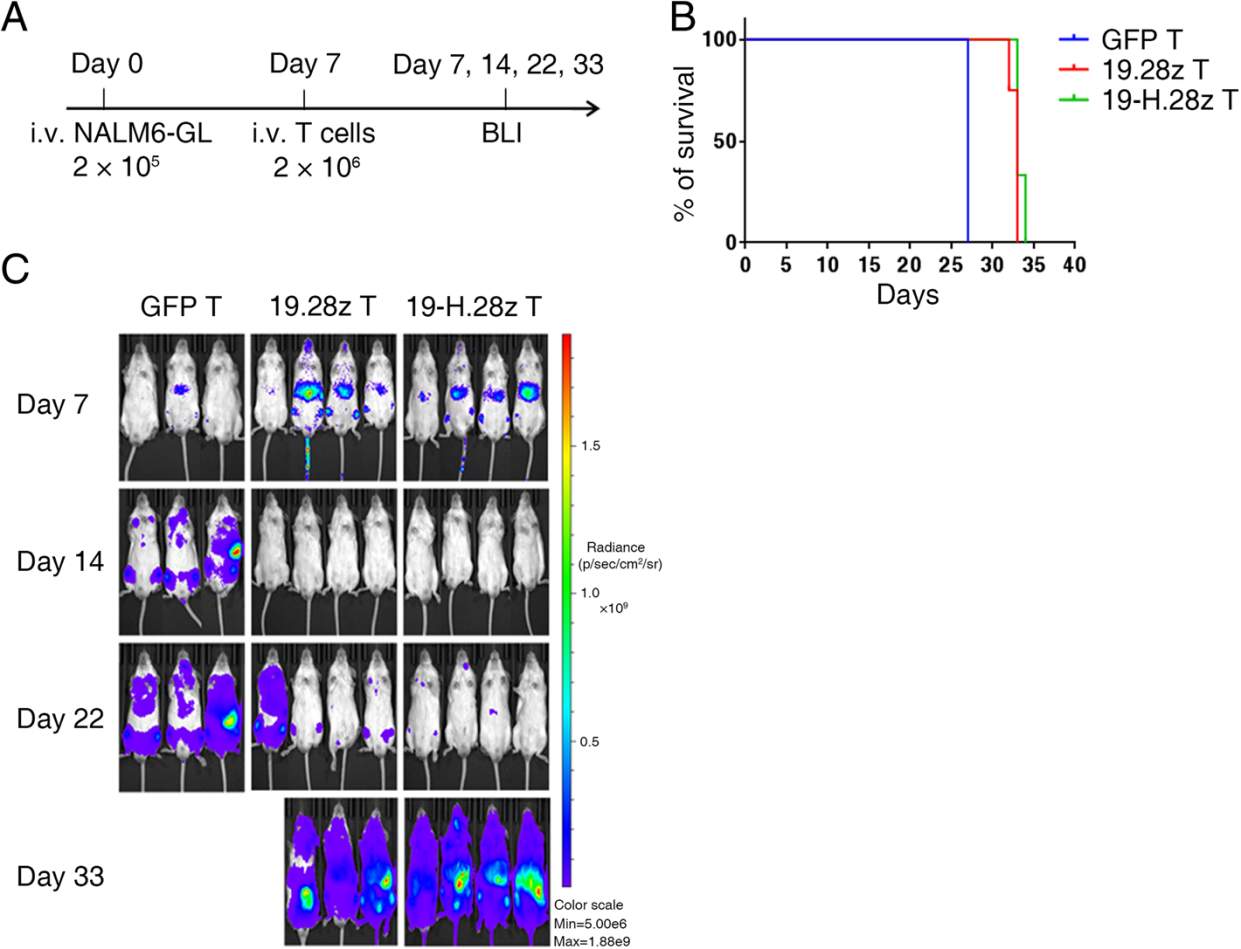
19-H.28z T and 19.28z T cells have comparable antitumor efficacy in vivo. **a** Timeline and events of the experiment with intravenous NALM6-GL xenograft models. On day 0, 2 × 10^5^ NALM6-GL cells were injected through the tail vein into the NSI mice, and on day 7, 2 × 10^6^ GFP T, 19.28z T or 19-H.28z T cells were injected through the tail vein into each NALM6-GL-NSI mouse. The cell numbers refer to transduced CAR T cells. *n* = 3 for the GFP T cell group, and *n* = 4 for the 19.28z T and 19-H.28z T cell group. On day 7, 14, 22, and 33, bioluminescence imaging was conducted. **b** Survival analysis of mice treated with GFP T, 19.28z T, or 19-H.28z T cells. **c** Bioluminescence images of mice treated with GFP T, 19.28z T, or 19-H.28z T cells on day 7, 14, 22, and 33 post-infusion of 2 × 10^5^ NALM6-GL cells

Subsequently, we evaluated the in vivo antitumor capacities of CAR T cells with or without the hinge domain in cell-line-derived xenograft-bearing mice. Immune deficient NSI mice were intravenously injected with 2 × 10^5^ NALM6-GL cells, followed by the infusion of a single dose of 2 × 10^6^ GFP, 19.28z, or 19-H.28z T cells on day 7 (Fig. 4a) [32–36]. Bioluminescence imaging (BLI) showed a reduced tumor burden in the mice infused with 19.28z T and 19-H.28z T cells compared with those infused with GFP T cells on day 14. However, both the 19.28z T and 19-H.28z T-cell-infused mice relapsed on day 22 (Fig. 4c). The survival times of the 19.28z T and 19-H.28z T cell groups also showed no significant difference (Fig. 4b). In summary, the introduction of a hinge into anti-CD19 specific CARs did not enhance their in vivo antitumor capacities.

### Meso-H.28z T cells exhibit enhanced antitumor capacities in vivo

**Fig. 5:**
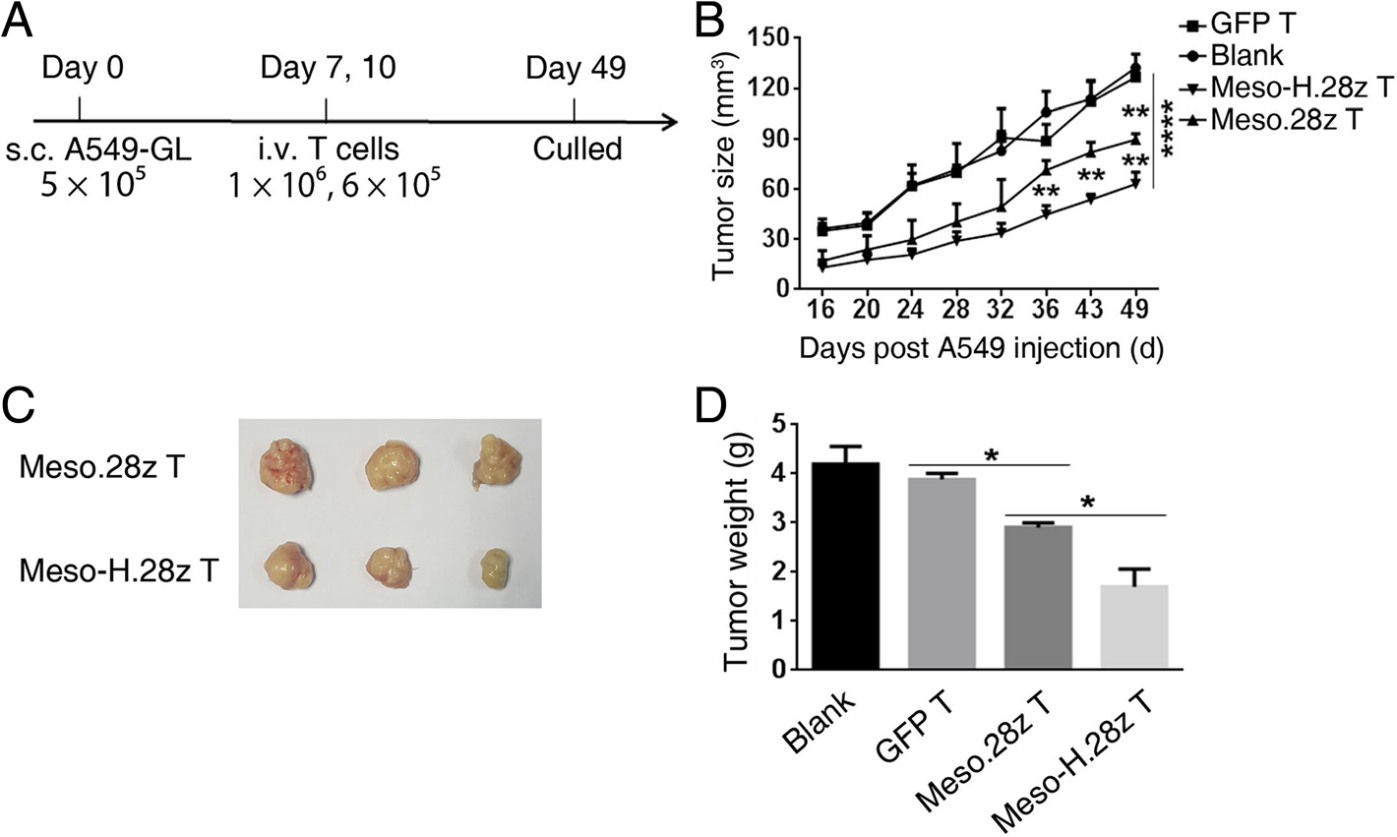
Meso-H.28z T cells exhibit enhanced antitumor capacities in vivo. **a** Timeline of events of the xenograft experiment with the subcutaneous inoculation of A549-GL cells. On day 0, 5 × 10^5^ A549-GL cells were injected subcutaneously into the NSI mice, and on day 7 and day 10, 1 × 10^6^ and 6 × 10^5^ GFP T, Meso.28z T or Meso-H.28z T cells were injected through the tail vein into each mouse (*n* = 3). The cell numbers refer to transduced CAR T cells. **b** Tumor burden of mice treated with GFP T, Meso.28z T, or Meso-H.28z T cells from day 16 to 49 after A549-GL cell inoculation. On day 49, the mice were sacrificed and the tumors were analyzed. **c**–**d** Size and weight of subcutaneous A549-GL tumors from NSI mice treated with GFP T, Meso.28z T, or Meso-H.28z T cells on day 49 post-inoculation of A549-GL cells. *Error bars* denote the s.e.m. and the groups were compared through an unpaired *t* test. *P < 0.05, **P < 0.01, and ***P < 0.001

To study whether a hinge can affect the antitumor capacities of CARs that are specific for solid tumors, a solid-tumor mouse model was established, in which A549-GL cells were subcutaneously injected into NSI mice. Mice bearing an established tumor were treated I.V. with two doses of Meso.28z, Meso-H.28z, or GFP T cells, with the first dose on day 7 and the second on day 10. The tumor diameters were measured every 6 days. On day 49, the mice were sacrificed (Fig. 5a). Interestingly, tumor growth in both the Meso.28z T and Meso-H.28z T cell groups was delayed compared with the blank and GFP T cell group, but the delay was greater in the Meso-H.28z T group (Fig. 5b). Results consistent with these were also obtained from weight measurement and photographic inspection of the tumors (Fig. 5c–d). These results demonstrated that the incorporation of a hinge domain enhanced the antitumor capacities of anti-mesothelin CARs.

## Discussion

Despite the remarkable progress in CAR T cell-based immune therapy, several obstacles remain [37, 38]. For example, the efficiency of CAR T cell expansion requires improvement. Recently, some groups have reported that an optimal CD4/CD8 ratio is important for the in vivo antitumor activity of CAR T cells, and the percentage of CD4+ CAR T cells is positively correlated with patient recovery rates [39–42]. Because CD8+ T cells tend to be preferentially expanded in current T cell in vitro culture systems [43], a method to promote the expansion of CD4+ T cells is urgently needed. Herein, we found that both IgG4-CH3 and IgD hinges were able to continuously increase the CAR T cell percentages and absolute cell numbers during the in vitro culture period [20, 44], and even more importantly, the additional CAR T cells were mainly CD4+. However, when we isolated the CD4+ T and CD8+ T cells and cultured them separately in vitro, the increased-growth effect disappeared. The mechanism of the increased growth will be studied by us in the future.

To date, many different versions of anti-CD19 CARs have been used in clinical trials [38]. The scFvs of these CARs are almost all derived from FMC63 mAb [45], while various different hinge domains and costimulatory molecules have been used. For example, CTL019, which is the most widely used CAR in CD19+ leukemia and lymphoma treatment, has a CD8α hinge [46, 47], while CD19RCD28 CAR uses a modified IgG4 hinge and Fc region [48], and also has a hinge-deleted version [19]. The functional differences between CD28 and 41-BB costimulatory molecules have already been well characterized [49]; however, the influence of the hinge domain on anti-CD19 CARs has not been studied. Our results showed that the killing activities of 19.28z T and 19-H.28z T cells were similar whether in vitro or in vivo. There are several possible explanations of this result: the location of the CD19 epitope recognized by the FMC63 mAb is not membrane-proximal, there are no steric inhibitory effects between FMC63 mAb and its epitope, or the density of the CD19 molecule on tumor cells is high [50]. These factors may also partially explain the greater popularity of CD19 in clinical trials compared with CD20 and CD22.

Mesothelin is a glycosyl–phosphatidyl inositol-linked cell surface glycoprotein, which is highly expressed in mesothelioma, and lung, pancreas, breast, ovarian, and other cancers, and has been used as a tumor antigen of CAR T cells in several trials [51]. We have shown that Meso-H.28z T cells have a better tumor-eradication capacity than Meso.28z T cells in tumor-bearing mice, suggesting that the mesothelin epitope recognized by the scFv may be membrane-proximal, or that there exist steric inhibitory effects between scFv and its epitope, like in SM3 mAb (a MUC1-specific mAb) [20]. Thus, a hinge is necessary to reduce the distance or ameliorate the steric inhibitory effects between the scFv and its epitope. Furthermore, a hinge can also improve the flexibility of the scFv, which may also be one of the reasons why Meso-H.28z T cells exhibit better anti-tumor activities than Meso.28z T cells. The importance of Ab flexibility has also been demonstrated in naive B cells which co-express cell surface IgM, which lacks a hinge, and IgD, whose elongated monomeric hinge is the longest of all Ab isotypes [52]. As a result, IgD can assume a “T shape” in which Fab regions can engage Ag in virtually any orientation, making the scFv omni-directional.

## Conclusions

To summarize, our data demonstrate that the incorporation of a hinge domain can enhance CAR T cell expansion during the in vitro culture period, mainly by promoting CD4+ CAR T cell proliferation, and a hinge domain can also enhance the antitumor efficacy of some specific CARs. Our results suggest potential novel strategies in CAR vector design.

## Additional files

Additional file 1: Figure S1. Hinge incorporation can promote the expansion of CAR T cells. Flow cytometric analysis of the percentage of 19.28z, 19-H.28z T cells, Meso.28z, Meso-H.28z T cells, PSCA.28z, PSCA-H.28z T cells, and GFP control T cells from day 6 to 15 during the in vitro culture period. The data are representative of independent experiments verified with cells from over three individual healthy human donors. (JPG 2876 kb)
Additional file 2: Figure S2. Hinge incorporation promotes CD4+ anti-CD19 CAR T cell expansion. Flow cytometric analysis of the percentage of CD4+ and CD8+ GFP T, 19.28z T, and 19-H.28z T during the in vitro culture period. The data are representative of independent experiments verified with cells from over three individual healthy human donors. (JPG 2320 kb)
Additional file 3: Figure S3. Percentages of CD4+ and CD8+ CAR T cells without a hinge domain both tended to be stable throughout the in vitro culture period. Flow cytometric analysis of the percentage of CD4+ and CD8+ (A) 19.28z T, (B) Meso.28z T, (C) PSCA.28z T, (D) HER2.28z T, and (E) GFP control T cells during the in vitro culture period. The data are representative of independent experiments verified with cells from over three individual healthy human donors. (JPG 924 kb)

## Abbreviations

BLI: Bioluminescence imaging
CAR: Chimeric antigen receptor
HER2: Human epidermal growth factor receptor 2
Meso: Mesothelin
PSCA: Prostate stem cell antigen
scFv: Single-chain fragment variable
