# Supplementary figures and images for: Incorporation of a hinge domain improves the expansion of chimeric antigen receptor T cells
Source: J Hematol Oncol. 2017 Mar 13;10:68. doi: 10.1186/s13045-017-0437-8 (PMC5347831; doi:10.1186/s13045-017-0437-8)

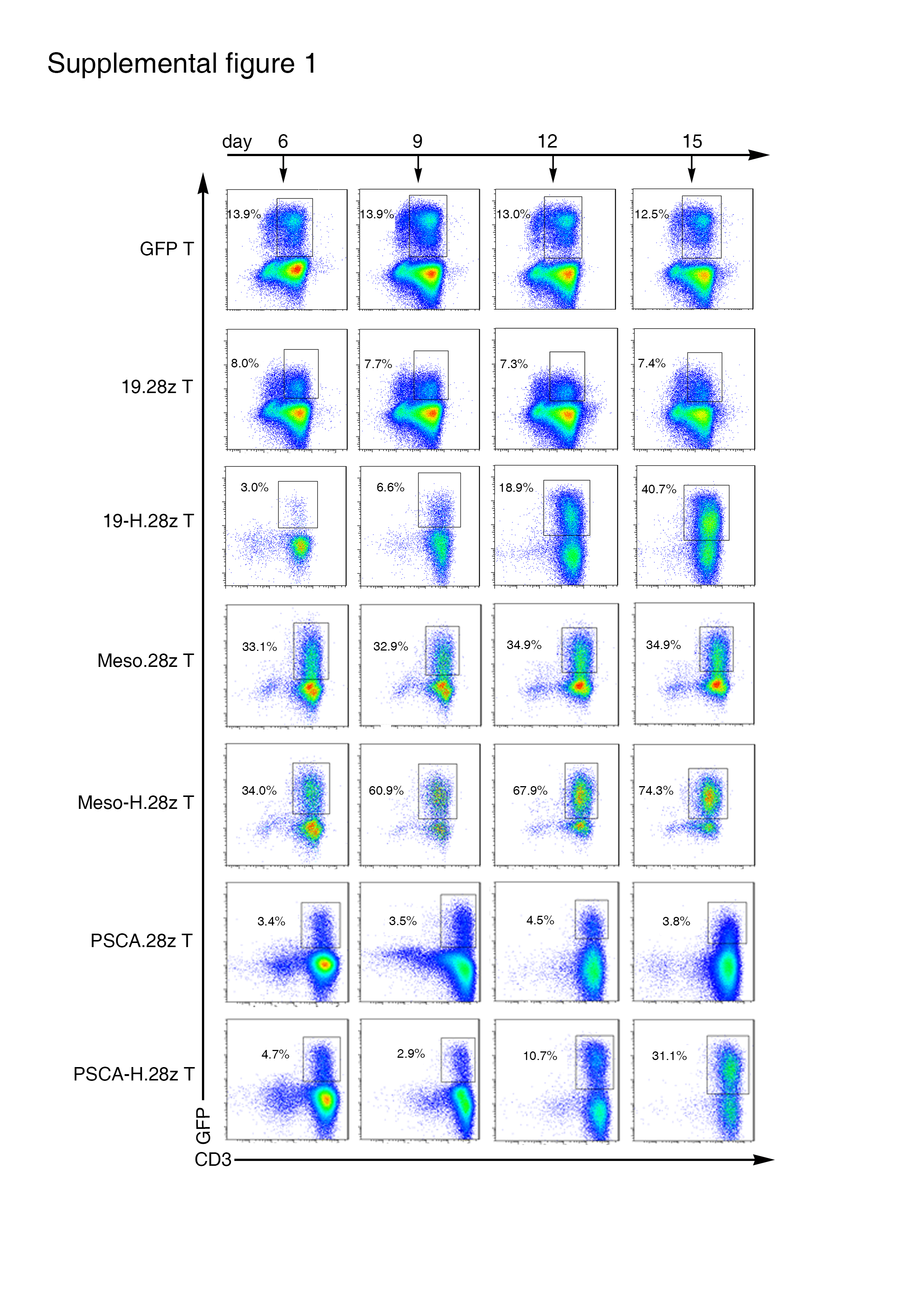

Supplement: Additional file 1: Figure S1. — Hinge incorporation can promote the expansion of CAR T cells. Flow cytometric analysis of the percentage of 19.28z, 19-H.28z T cells, Meso.28z, Meso-H.28z T cells, PSCA.28z, PSCA-H.28z T cells, and GFP control T cells from day 6 to 15 during the in vitro culture period. The data are representative of independent experiments verified with cells from over three individual healthy human donors. (JPG 2876 kb) [file 13045_2017_437_MOESM1_ESM.jpg]

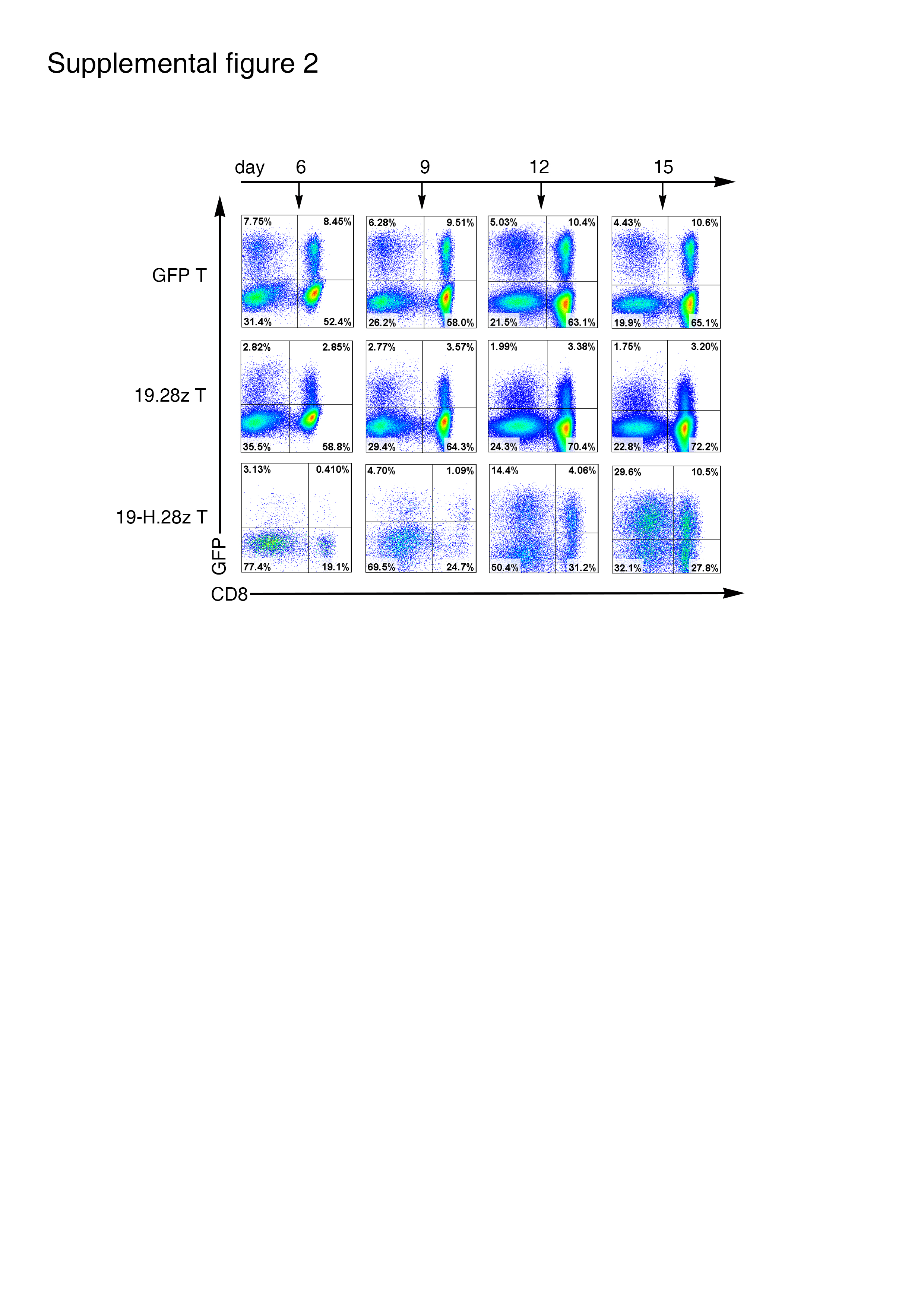

Supplement: Additional file 2: Figure S2. — Hinge incorporation promotes CD4+ anti-CD19 CAR T cell expansion. Flow cytometric analysis of the percentage of CD4+ and CD8+ GFP T, 19.28z T, and 19-H.28z T during the in vitro culture period. The data are representative of independent experiments verified with cells from over three individual healthy human donors. (JPG 2320 kb) [file 13045_2017_437_MOESM2_ESM.jpg]

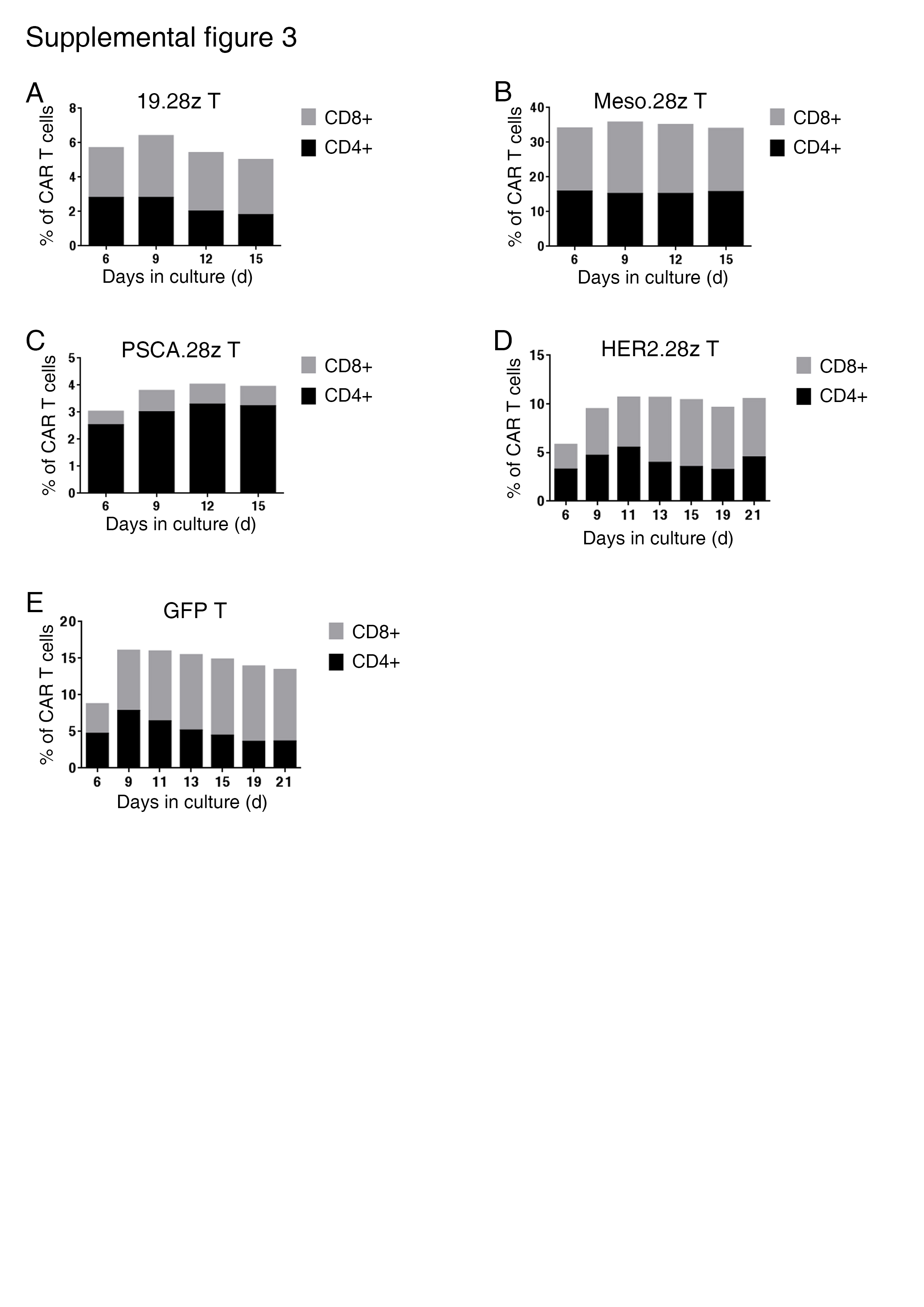

Supplement: Additional file 3: Figure S3. — Percentages of CD4+ and CD8+ CAR T cells without a hinge domain both tended to be stable throughout the in vitro culture period. Flow cytometric analysis of the percentage of CD4+ and CD8+ (A) 19.28z T, (B) Meso.28z T, (C) PSCA.28z T, (D) HER2.28z T, and (E) GFP control T cells during the in vitro culture period. The data are representative of independent experiments verified with cells from over three individual healthy human donors. (JPG 924 kb) [file 13045_2017_437_MOESM3_ESM.jpg]
